# The galanin system in depression and antidepressant treatment: focus on the locus coeruleus

**DOI:** 10.1186/2050-6511-13-S1-A75

**Published:** 2012-09-17

**Authors:** Simone B Sartori, Anupam Sah, Baosheng Zhao, Inga Neumann, Rainer Landgraf, Nicolas Singewald

**Affiliations:** 1 Department of Pharmacology and Toxicology, University of Innsbruck, 6020 Innsbruck, Austria; 2 Department of Zoology, University of Regensburg, 93040 Regensburg, Germany; 3 Max-Planck Institute of Psychiatry, 80804 Munich, Germany

## Background

Our knowledge about central changes underlying depressive disorders is still incomplete, but disturbances in monoaminergic neurotransmission are involved. There is also increasing evidence for a possible role of the neuropeptide galanin and its three G protein-coupled receptors in the pathophysiology and treatment of depression [1].

## Methods

Using *in situ* hybridization we investigated whether transcriptional processes in the galanin system may be involved in the heightened depression-like behaviour of HAB rats selectively bred for high trait anxiety as compared with their low anxiety/depression (LAB) counterparts [2] and in the treatment responses to established antidepressant drugs. Subsequently, the modulation of depression-related behaviour by intra-cerebrally applied galaninergic ligands was studied in HAB and LAB rats.

## Results

The abundance of galanin mRNA was increased in the paraventricular hypothalamus, the central amygdala and the locus coeruleus (LC), but not in the dorsal raphe of HAB as compared to LAB animals. Conversely, long-term (42 days, p.o.) treatment with either desipramine, paroxetine or tranylcypromine caused a general reduction in galanin mRNA expression in the locus coeruleus (LC) of unselected rats indicating a common response to antidepressant drug treatment while in the paraventricular hypothalamus galanin mRNA was increased by tranylcypromine only. This observed common effect of the antidepressants on galanin mRNA in the LC is in contrast to the finding in the HAB model raising the exciting possibility that altered coerulear galanin mRNA expression may be associated with depression-related behaviour. Indeed, intra-LC galanin caused a pronounced increase in the immobility of LAB rats indicating enhanced depression-like behaviour while a galanin receptor antagonist reduced immobility in HAB rats.

## Conclusions

The present data suggest that depression-like behaviours can be altered by interference with the galanin system and, thus, the galanin system may represent an interesting target for novel antidepressant pharmacotherapy. In particular, its modulation in the LC, where galanin highly coexists with noradrenaline, appears to be critical.
